# High-Altitude Pulmonary Embolism: Epidemiology, Pathophysiology, Diagnosis, and Management

**DOI:** 10.1155/carj/5519627

**Published:** 2025-09-15

**Authors:** Zhen-Zhong Yang, Jing Li, Zhen-Long Chang, Xiao-Xia Liu, Jun-Peng Ran, Lin-Feng Liu

**Affiliations:** ^1^Emergency Department, People's Hospital of Xizang Autonomous Region, Lhasa 850000, China; ^2^Cardiovascular Department of Mountain Diseases, People's Hospital of Xizang Autonomous Region, Lhasa 850000, China; ^3^Emergency Department of People's Hospital of Lhasa, Lhasa 850000, Xizang Autonomous Region, China; ^4^Department of Anesthesia and Critical Care, The Second Affiliated Hospital and Yuying Children's Hospital of Wenzhou Medical University, Wenzhou 325027, China

**Keywords:** diagnosis, high-altitude, pathophysiology, pulmonary embolism, VTE

## Abstract

Pulmonary embolism (PE) at high altitude (HA) is a potentially life-threatening but underrecognized condition. Unlike low-altitude PE, high-altitude pulmonary embolism (HA-PE) may result from unique hypoxia-driven mechanisms, including hemoconcentration, endothelial dysfunction, and a hypercoagulable state. In this narrative review, we summarize current evidence on the epidemiology, pathophysiology, diagnosis, management, and prognosis of HA-PE, based on the literature published between 2010 and 2025 retrieved from PubMed and CNKI. This review summarizes the epidemiological profile, clinical features, altitude-related diagnostic challenges, limitations of current therapeutic strategies, and the prognosis of HA-PE. A more comprehensive understanding of HA-PE is crucial for enhancing early detection and developing altitude-adapted management approaches.

## 1. Introduction

Pulmonary embolism (PE) is a life-threatening manifestation of venous thromboembolism (VTE) and ranks as the third most common acute cardiovascular disorder worldwide, following myocardial infarction and stroke [1, 2]. The global burden of PE is increasing, with significant implications for public health and clinical management. High-altitude (HA) environments (≥ 2500 m) are characterized by hypobaric hypoxia, low atmospheric pressure, cold temperatures, and intense solar radiation, posing complex physiological challenges [3, 4]. With the growing number of individuals exposed to such environments—including military personnel, miners, mountaineers, and native residents—the population at risk of PE under HA conditions is expanding. However, the unique thrombotic risks in these settings remain underrecognized and poorly studied

Most studies on PE to date have centered on low-altitude populations, with current guidelines failing to consider the unique epidemiology, mechanisms, and clinical challenges of PE at HAs [21, 22]. High-altitude pulmonary embolism (HA-PE) has been reported in isolated cases, but a systematic review is still lacking. As a form of VTE [23], HA-PE shares core features such as thrombus formation and pulmonary artery obstruction. However, its occurrence under hypobaric hypoxia may promote erythrocytosis, hemoconcentration, endothelial dysfunction, and inflammatory activation, contributing to a distinct prothrombotic state [24]. HA-PE may therefore represent a context-specific VTE subtype with unique epidemiological and clinical characteristics that warrant further investigation. This review aims to synthesize current evidence across six key domains: epidemiology, pathophysiology, classification, clinical presentation, diagnosis and differential diagnosis, and prognosis, to improve understanding of HA-PE in HA settings.

To ensure the systematicity and representativeness of this review, a literature search was conducted in the PubMed and CNKI databases for articles published between 2010 and 2025. The search terms included “pulmonary embolism,” “venous thromboembolism,” “high altitude,” “hypoxia,” and “thrombosis.” Both English and Chinese publications were considered. Eligible studies included original research related to HA-PE or deep vein thrombosis (DVT), such as case reports and cohort studies. Studies not involving HA settings or lacking sufficient clinical detail were excluded.

## 2. Epidemiology of HA-PE and VTE

Although PE, as a major clinical manifestation of VTE, has long been widely recognized, most attention has traditionally focused on established risk factors, such as surgery, immobility, malignancy, advanced age, obesity, and pregnancy [25–29]. HA exposure, however, as a potential environmental risk factor, has not been sufficiently appreciated. In recent years, accumulating evidence from retrospective cohorts, prospective studies, and case reports has suggested that HA environments may significantly increase the risk of VTE, particularly PE. Our synthesis of 16 original studies involving diverse populations—including military personnel, mountaineers, postoperative orthopedic patients, and long-term HA residents—indicates a generally elevated VTE risk above 4000 feet.

In recent years, an increasing number of studies have indicated that HA environments are significantly associated with an elevated risk of postoperative VTE, particularly PE (see Table 1). In a retrospective study involving 29,827 patients undergoing arthroscopic rotator cuff repair, Cancienne et al. reported that the 90-day incidence of PE was markedly higher at elevations ≥ 4000 feet compared to lower altitudes (OR = 4.3, *p* < 0.0001), with a similarly increased risk of DVT (OR = 2.2, *p* < 0.029) [8]. Donnally et al. reported a 90-day PE incidence of 0.49% after lumbar fusion at HA, higher than rates observed at low altitude [5]. Jones et al. observed that patients with tibial plateau fractures experienced significantly increased 90-day postoperative risks of DVT (OR = 1.21, *p*=0.043) and PE (OR = 1.27, *p*=0.037) at HAs [12]. In patients undergoing total knee arthroplasty (TKA), Plancher et al. demonstrated a significantly higher incidence of VTE in the HA group at both 30 days (OR = 1.15, *p*=0.022) and 90 days (OR = 1.20, *p*=0.00007) postoperatively [19]. Additionally, in a large-scale cohort of 458,655 patients undergoing meniscectomy or chondroplasty, Cancienne et al. reported significantly elevated 30-day VTE (OR = 2.0, *p*=0.0003) and PE (OR = 2.5, *p*=0.0099) rates following procedures at HA compared to low altitude [20]. Collectively, these findings across various surgical contexts consistently suggest that HA exposure may serve as an independent risk factor for postoperative VTE, particularly PE, underscoring the need for heightened perioperative risk assessment and targeted preventive strategies in such environments.

HA exposure—whether short-term or chronic—has been increasingly recognized as a nontraditional but important risk factor for VTE, particularly PE. In the context of short-term exposure, several studies have reported PE cases among healthy individuals temporarily deployed to elevations ranging from 3000 to 5300 m. Most events occurred within days to months following ascent, highlighting early altitude adaptation as a potential window of thrombotic vulnerability. At the Military Hospital in Rawalpindi, Pakistan, Khalil et al. found that nearly half of 50 soldiers with suspected PE had no identifiable risk factors other than recent altitude exposure, implicating hypobaric hypoxia as a potential primary trigger [30]. Similarly, a retrospective study by Xiong et al. (2004–2022) in Yecheng County, China, showed a markedly increased risk of DVT among HA residents (OR = 16.3; *p*=0.036), particularly during early exposure to extreme elevations [11]. An Indian military cohort study reported 44 idiopathic PE cases and 2 VTE events among 53 soldiers stationed at altitudes of 10,000–22,000 feet during a 6-month deployment [14]. In a large-scale prospective study, Nair et al. observed a VTE incidence of 5926 per 100,000 person-years and a DVT ± PE incidence of 2469 per 100,000 person-years among 960 climbers exposed to elevations ≥ 15,000 feet (approximately 4572 m), far exceeding background rates in the general population [7]. In terms of chronic exposure, a 2-year prospective study conducted in Saudi Arabia found that individuals residing permanently at HAs had significantly higher rates of VTE compared to low-altitude residents, with DVT incidence of 56.8% versus 13.0%, and PE incidence of 12.6% versus 4.1% [17].

As a reference, in low-altitude populations, the annual incidence of PE is approximately 39–115 per 100,000 persons, while that of DVT ranges from 53 to 162 per 100,000 persons [31, 32]. In Europe alone, estimated 370,000 deaths were attributed to VTE in 2004, among a total population of approximately 450 million, with about 34% of fatal PEs occurring within hours of symptom onset—highlighting the acute and life-threatening nature of the condition [33]. Although existing HA studies vary in design, population characteristics, and diagnostic modalities—and no unified incidence estimate has been established—the overall data suggest a substantially elevated risk of VTE, particularly PE, in HA settings. The cumulative evidence supports the notion that HA exposure may represent a potential independent risk factor for PE. This underscores the need for heightened clinical vigilance in such environments and calls for further high-quality prospective studies to confirm this association.

## 3. Mechanisms of HA-PE

**Figure 1:**
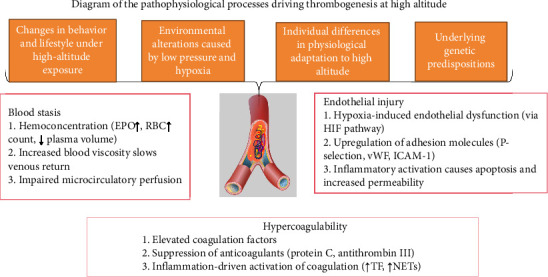
Pathopysiological processes driving thrombogenesis at high altitude. EPO, erythropoietin; RBC, red blood cell; HIF, hypoxia-inducible factor; TF, tissue factor; NETs, neutrophil extracellular traps; vWF, von Willebrand factor; ICAM-1, intercellular adhesion Molecule-1; Antithrombin III, a natural anticoagulant; protein C, a vitamin K–dependent anticoagulant protein.

HA-PE represents a severe clinical manifestation of VTE under HA conditions. Its underlying pathophysiological mechanisms are consistent with those of VTE in general, centering on the three components of Virchow's triad—venous stasis, hypercoagulability, and endothelial injury—ultimately leading to pulmonary vascular involvement [34, 35]. However, the unique environmental stressors at HA, particularly hypobaric hypoxia, markedly amplify each axis of this triad, thereby enhancing thrombotic risk and often resulting in more abrupt onset and pronounced clinical manifestations of PE (see Figure 1).

The hypoxic environment at HA stimulates the secretion of erythropoietin (EPO), promoting increased erythropoiesis [36]. This process, coupled with reduced plasma volume, leads to typical hemoconcentration and increased blood viscosity, commonly referred to as “HA hyperviscosity.” [37] Such changes are frequently observed in individuals with prolonged HA exposure or those engaging in intense physical activity, significantly elevating blood viscosity. Increased blood viscosity elevates vascular resistance and slows venous blood flow, particularly in the deep veins of the lower extremities, leading to venous stasis and localized hypoxia—factors that significantly enhance the risk of thrombus formation. At the same time, elevated shear stress exerts mechanical injury on the vascular endothelium, activates oxidative stress pathways, and disrupts the dynamic balance between anticoagulant and fibrinolytic systems, further promoting a prothrombotic state [38, 39] (see Figure 1).

**Figure 2:**
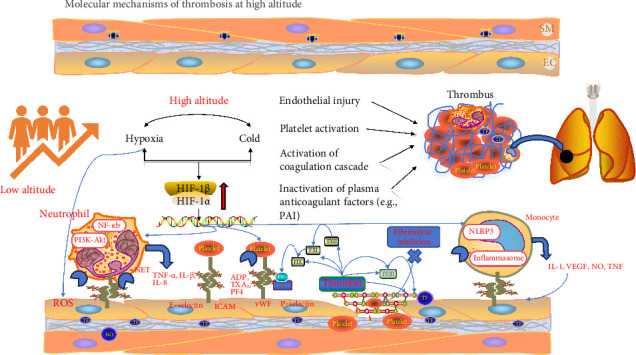
High-altitude exposure to hypoxia and cold stabilizes hypoxia-inducible factors (HIF-1*α*/1β), activating transcriptional programs that disrupt vascular integrity, promote inflammation, and enhance coagulation. Hypoxic endothelial cells undergo oxidative damage and release adhesion molecules (e.g., P-selectin, ICAM-1, and vWF), facilitating leukocyte and platelet adhesion. Meanwhile, hypoxia induces neutrophil extracellular trap (NET) formation and NLRP3 inflammasome activation in monocytes, driving the release of proinflammatory cytokines (e.g., IL-1, TNF, and VEGF). These processes collectively trigger platelet activation and coagulation cascades (TF, FXII, FXI, FX, FVIII, and FXIII), while suppressing natural anticoagulants (protein C and Antithrombin III) and impairing fibrinolysis, ultimately leading to thrombus formation and pulmonary artery obstruction. Abbreviations: HIF, hypoxia-inducible factor; TNF-α, tumor necrosis factor alpha; IL, interleukin; ICAM-1, intercellular adhesion Molecule-1; PAI: plasminogen activator inhibitor; vWF, von Willebrand factor; NETs, neutrophil extracellular traps; TF, tissue factor; FXII, FXI, FX, FVIII, and FXIII, coagulation factors XII, XI, X, VIII, and XIII; VEGF, vascular endothelial growth factor; NLRP3, NOD-like receptor family pyrin domain containing 3.

The hypoxic or cold environment at HA induces endothelial dysfunction through multiple converging pathways, thereby promoting thrombogenesis and establishing a hypercoagulable state characterized by oxidative stress, inflammatory activation, and coagulation–fibrinolysis imbalance [40, 41]. First, hypoxia activates the hypoxia-inducible factor (HIF) (HIF-1α/HIF-1β) signaling pathway, leading to excessive production of reactive oxygen species (ROS), which depletes nitric oxide (NO), impairs vasodilation, and enhances platelet adhesion and aggregation [42]. ROS also directly damages endothelial cells, increasing vascular permeability and providing a substrate for subsequent inflammatory and procoagulant responses. Second, hypoxia activates inflammatory pathways such as NF-κB, stimulating neutrophils and monocytes to release proinflammatory cytokines, including IL-6 and TNF-α [43, 44]. Meanwhile, the expression of von Willebrand factor (vWF) is upregulated, exacerbating endothelial adhesiveness and procoagulant potential, thereby amplifying thrombotic signaling cascades. Finally, hypoxia suppresses endogenous anticoagulant and fibrinolytic mechanisms: the expression of protein C, prostacyclin (PGI_2_), and tissue factor pathway inhibitor (TFPI) is reduced, while plasminogen activator inhibitor-1 (PAI-1) is upregulated, inhibiting fibrinolysis. In parallel, tissue factor (TF) expression is increased, activating the extrinsic coagulation pathway and promoting thrombin generation and fibrin deposition, thus accelerating clot formation and enhancing its stability [45] (see Figure 2).

Platelets play an important role in the early phases of thrombus formation by interacting with innate immune cells such as neutrophils and monocytes, contributing to thrombus stabilization and propagation. These interactions—partially mediated by adhesion molecules like GPIbα—facilitate platelet aggregation and may enhance neutrophil activation, including the release of neutrophil extracellular traps (NETs), which together promote a localized prothrombotic and proinflammatory environment [46].

Recent transcriptomic studies indicate that HA thrombosis is not solely driven by hypoxic exposure but also involves distinct gene–environment interactions. Jha et al. identified altitude-specific differentially expressed genes enriched in pathways related to platelet function, coagulation cascades, and hypoxia responses. These findings suggest that hypobaric hypoxia may transcriptionally reprogram platelet activity, leading to a prothrombotic phenotype distinct from that observed at sea level [47].

## 4. Clinical Phenotypes of HA-PE

In recent years, research on VTE and PE in HA environments has increased significantly. While most studies to date have focused on incidence, risk factors, and epidemiological characteristics across specific populations, few have attempted to mechanistically stratify HA-PE. Building upon existing findings and current understanding of hypoxia-induced thrombogenesis, we propose a preliminary mechanism-driven classification of HA-PE into three clinical phenotypes: (1) short-term exposure (e.g., mountaineers, short-term travelers), (2) long-term sojourners (e.g., military personnel, construction workers), and (3) HA permanent residents.

Individuals with short-term HA exposure, lacking effective acclimatization, are subjected to acute hypoxia and fluctuating pulmonary hypertension. These physiological stressors lead to heterogeneous pulmonary vasoconstriction, ventilation–perfusion (V/Q) mismatch, and impaired oxygen transport [48, 49]. Sympathetic nervous system activation, increased circulatory load, strenuous exertion, and dehydration further amplify Virchow's triad, substantially elevating thrombosis risk. Clinically, HA-PE in this context presents acutely with chest pain, dyspnea, and hypoxemia—often mimicking high-altitude pulmonary edema (HAPE)—and may be misdiagnosed, delaying anticoagulant therapy. A study by Yanmin Liu et al. showed that 38.9% of severe HAPE patients had concurrent VTE, which was associated with prolonged hospitalization, increased respiratory failure, and elevated D-dimer levels, indicating that VTE is a critical determinant of adverse outcomes in this setting [9]. Mechanistically, short-term HA exposure is associated with a pronounced procoagulant and hypofibrinolytic state. In a prospective study of 960 mountaineers, Nair et al. reported that individuals exposed to altitudes ≥ 4572 m exhibited significantly elevated levels of fibrinogen (Fbg), D-dimer, PAI-1, and plasminogen (PLG), alongside reduced levels of tissue plasminogen activator (tPA) (all *p* < 0.05), indicating a classic fibrinolytic suppression profile. Individuals who developed thrombotic events also showed increased thrombin generation (FVIIa and FXa ↑, *p* < 0.001), suppressed natural anticoagulant activity (Thrombomodulin ↓, TFPI ↓), and a downward trend in tPA (*p*=0.078). In parallel, markers of endothelial dysfunction and systemic inflammation—such as VCAM-1, ICAM-1, VEGFR-3, P-selectin, CD40L, CRP, and MPO—were significantly elevated (all *p* < 0.001), suggesting that hypoxia promotes thrombogenesis via a multifaceted mechanism involving coagulation activation, anticoagulant suppression, impaired fibrinolysis, and endothelial inflammation [7]. Similarly, in a study of 226 participants—including healthy HA residents and low-altitude controls—Jiang et al. found that plasma levels of Fbg, D-Di, PAI-1, and PLG were significantly higher in the HA group, while tPA was significantly lower (all *p* < 0.05), reinforcing the presence of a chronic hypercoagulable and hypofibrinolytic state under prolonged hypoxic exposure [50].

Individuals with prolonged HA residence—such as stationed military personnel, construction workers, and researchers—typically remain at altitude for several weeks to months. Although partial acclimatization may occur, chronic hypoxic exposure can still lead to sustained pulmonary vasoconstriction and vascular remodeling [51], resulting in elevated pulmonary artery pressure (classified as Group 3 PH in the clinical classification) [52] and increased right ventricular afterload. More importantly, these individuals often experience a semi-sedentary lifestyle during their stay, especially in cold climates or construction settings, where prolonged sitting, delayed urination, and dehydration are common, all of which contribute to venous stasis and heightened thrombotic risk [53]. Additionally, the use of sedatives or diuretics to alleviate altitude sickness or improve sleep may further disrupt hemodynamic stability and coagulation balance. Clinically, PE in this population tends to have an insidious onset with atypical early symptoms, such as mild exertional dyspnea, fatigue, or signs of DVT, and is often detected incidentally or after clinical deterioration. A cross-sectional study by Cai et al. demonstrated that long-term HA residents commonly exhibit biochemical features such as hypoxemia, elevated hemoglobin levels, and increased D-dimer concentrations, suggesting a typical hypercoagulable state and underscoring the need for increased vigilance regarding thrombotic risk in this population [16].

Indigenous HA residents—such as Tibetans on the Qinghai–Tibet Plateau, Himalayan populations, and Andean natives—exhibit certain physiological adaptations to chronic hypoxia, including elevated hemoglobin levels, enhanced pulmonary vasodilation responses, and genetic polymorphisms in the HIF signaling pathway. However, such adaptations do not confer absolute protection against thrombosis. With increasing age, the accumulation of metabolic disorders (e.g., hypertension, diabetes, dyslipidemia), decreased physical activity, and high-fat, high-salt dietary patterns, the risk of VTE among these populations also rises. Studies have shown that some native HA residents present with a hypercoagulable state, hemorheological alterations, and reduced fibrinolytic activity, suggesting the emergence of a novel thrombosis-prone phenotype under chronic hypoxic exposure. Importantly, PE in this population often occurs on the background of chronic comorbidities [54], with insidious onset and absence of typical symptoms such as chest pain or dyspnea, making misdiagnosis as HA heart disease or respiratory failure common. Accurate diagnosis thus relies on high clinical suspicion and appropriate auxiliary testing. Although PE has been infrequently reported among native HA residents, evidence from community-based epidemiological surveys—such as studies conducted in Abha, Saudi Arabia—suggests that its occurrence should not be overlooked [17].

In summary, individuals exposed to HA environments—including short-term visitors, long-term sojourners, and native residents—may exhibit distinct VTE and PE risk profiles due to varying physiological stressors and adaptive responses. However, systematic comparisons between HA and low-altitude populations regarding the incidence, clinical presentation, and underlying mechanisms of thromboembolic diseases remain limited. This study aims to compare the epidemiological characteristics, clinical manifestations, treatment strategies, and outcomes of VTE and PE between HA and low-altitude populations, in order to elucidate the potential impact of elevation on thrombotic risk and disease progression (see Tables 2 and 3).

## 5. Diagnosis and Differential Diagnosis of HPE

The diagnosis of HA-PE presents several challenges due to its nonspecific symptoms, altitude-induced physiological alterations, and overlap with other HA illnesses. Clinically, HA-PE often mimics conditions such as HAPE, pneumonia, and acute coronary syndrome, particularly in resource-limited settings, leading to frequent misdiagnoses or delayed recognition (see Table 4). At HAs, hypoxia can cause nonspecific elevations in D-dimer, which, while helpful in ruling out PE [55], has reduced diagnostic specificity. Similarly, biomarkers like high-sensitivity troponin (hs-cTn) and brain natriuretic peptide (BNP) may be elevated due to hypoxia-induced myocardial stress, limiting their utility in assessing PE severity [56, 57]. Furthermore, HA-PE frequently coexists with other altitude-related complications—such as pulmonary infections, right heart strain, or HA cerebral edema—complicating the diagnostic picture. Isolated PE without overt evidence of DVT also appears more common in HA populations, underscoring the need for a broader diagnostic perspective. Given these complexities, the diagnosis of PE at HA requires a context-specific, multiparametric approach that integrates clinical evaluation, imaging (CTPA), and dynamic monitoring—while accounting for the physiological impact of hypobaric hypoxia—to enhance diagnostic accuracy and timely intervention.

## 6. Treatment and Prognosis of HPE

The treatment principles for HA-PE generally follow standard PE management protocols [22], including early anticoagulation, oxygen therapy, and thrombolytic intervention when indicated. However, the HA environment poses unique physiological and logistical challenges—such as hypobaric hypoxia, limited access to imaging and laboratory diagnostics, and delays in patient transfer—that may result in delayed diagnosis and intervention, ultimately affecting prognosis. Notably, thrombolytic therapy must be administered with particular caution, as hypoxic stress may increase bleeding risk, while most HA settings lack advanced critical care monitoring facilities.

Currently, a few studies have addressed VTE prevention strategies specifically for HA regions. Nevertheless, emerging evidence suggests the need for individualized prophylactic plans for high-risk populations, such as mountaineers, military personnel, and postoperative bedridden patients. On-site preventive recommendations include maintaining adequate hydration, avoiding prolonged immobility, gradual altitude acclimatization, and early recognition of thrombosis-related symptoms. For high-risk individuals, pharmacologic prophylaxis with low-molecular-weight heparin (LMWH) or direct oral anticoagulants (DOACs) may be considered during prolonged HA stays or perioperative periods, although large-scale clinical trial evidence remains lacking.

The long-term prognosis of HA-PE has not been systematically characterized. However, some studies indicate that persistent hypoxia and treatment delays may contribute to chronic pulmonary hypertension, reduced exercise tolerance, and recurrent thrombotic events in a subset of patients [58, 59]. Whether long-term outcomes of HA-PE differ significantly from those at lower altitudes remains uncertain. Further research is urgently needed to identify altitude-related prognostic factors, including the incidence of chronic thromboembolic pulmonary hypertension (CTEPH) and the impact of prolonged altitude exposure on disease progression.

In summary, although the acute management of HA-PE aligns with conventional PE treatment principles, challenges related to diagnostic delay, prophylactic strategies for high-risk groups, and long-term cardiopulmonary sequelae warrant increased clinical attention. Altitude-specific approaches should be developed to optimize care in HA environments.

## 7. Conclusion

HA-PE represents a distinct clinical entity shaped by the interplay between environmental hypoxia and individual susceptibility. Across various exposure contexts—including acute visitors, long-term sojourners, and native highlanders—HA-PE may arise through hypoxia-induced endothelial dysfunction, coagulation imbalance, and impaired fibrinolysis. Diagnosis remains particularly challenging in resource-limited HA settings, where nonspecific symptoms and limited access to advanced imaging often lead to delays. While the principles of acute-phase management largely mirror those at sea level, therapeutic decision-making must account for altitude-specific physiological constraints and logistical barriers. Preventive strategies tailored to high-risk groups are currently lacking, and data on long-term outcomes—such as the risk of CTEPH—remain sparse. Moving forward, altitude-specific diagnostic algorithms, risk stratification tools, and evidence-based preventive interventions are urgently needed to improve clinical outcomes for HA-PE patients.

## Figures and Tables

**Table 1:** Reported cases and studies of venous thromboembolism (VTE) and pulmonary embolism (PE) at high altitude.

Author (year)	Study type	Sample size/case(s)	Altitude (feet)	PE status and diagnostic modality	VTE diagnosis status	Population/setting	Incidence or risk conclusion
Donnally et al. [5] (2019)	Retrospective study	High-altitude: Low-altitude: 44,289: 44,289	≥ 4000	Confirmed, diagnosed using ICD codes	NO	Patients undergoing lumbar fusion surgery	90-day incidence of postoperative pulmonary embolism: 0.49% [5]

Hull et al. [6] (2016)	Case report	A case	≥ 11,483	Confirmed, pulmonary CT diagnosis	Yes	Mountaineers	[6]

Nair et al. [7] (2022)	Prospective longitudinal study	960	≥ 15,000	Confirmed, pulmonary CT scan	Yes	Short-term mountaineers	VTE incidence: 5926 per 100,000 persons; DVT ± PTE incidence: 2469 per 100,000 person-years [7]

Cancienne et al. [8] (2016)	Retrospective study	29,827	> 4000	Confirmed, pulmonary CT scan	Yes	Postarthroscopic rotator cuff repair	Significantly increased 90-day incidence of PE (OR = 4.3) Postoperative DVT risk also elevated within 90 days (OR = 2.2) [8]

Liu et al. [9] (2023)	Retrospective study	18	> 12,139	Confirmed, CTPA	Yes	Severe high-altitude pulmonary edema	Increased VTE incidence in HAPE patients [9]

Wu et al. [10] (2025)	Retrospective study	79	> 16,404	Confirmed, CTPA	NO	17 subjects from extremely high-altitude training missions and 62 lowland controls	High-altitude exposure may increase susceptibility to PE in young individuals [10]

Xiong et al. [11] (2024)	Retrospective study	72	> 14,764	Confirmed, CTPA	Yes	HA residents (∼4900 ft) and very HA-exposed (> 14800 ft)	Higher PE incidence at high altitude Similar DVT rates between groups [11]

Jones et al. [12] (2022)	Retrospective study	7832	> 4000	Confirmed, CTPA	Yes	Patients with tibial plateau fractures at high and low altitudes	Higher altitude was associated with increased 90-day postoperative risk of DVT (OR 1.21, *p*=0.043) and PE (OR 1.27, *p*=0.037) [12]

Wu et al. [13] (2021)	Retrospective study	7	> 16,404	Confirmed, CTPA	Yes	Personnel stationed at extremely high altitudes	Pulmonary embolism is common in extremely high-altitude areas [13]

Dutta et al. [14] (2018)	Retrospective study	53	> 10,000	Confirmed, CTPA	Yes	Military personnel exposed to high-altitude hypoxia	Seventeen percent of patients had a hereditary predisposition to pulmonary embolism, while 83% presented with idiopathic PE [14]

Lu et al. [15] (2022)	Cross-sectional study	392	> 8200	Confirmed, CTPA	No	Hospitalized patients who have resided at high altitude continuously for ≥ 1 year	The characteristics of high-altitude pulmonary embolism patients vary by risk stratification [15]

Cai et al. [16] (2019)	Cross-sectional study	106	> 8858	Confirmed, CTPA	No	Hospitalized patients residing long term at high altitude	Patients with long-term residence at high altitude present with more pronounced hypoxemia, elevated hemoglobin concentrations, and increased D-dimer levels [16]

Algahtani et al. [17] (2020)	Prospective cohort study	234	> 7218	High-resolution CT (HRCT) scan	Yes	Suspected PE or VTE patients	PE incidence is significantly higher at high altitudes (4.1% vs. 0.4%), as is VTE incidence (81.9% vs. 21.9%) [17]

Singhal et al. [18] (2016)	Case report	2	17,000	Confirmed, CTPA	No	Native residents living at high altitude	High-altitude residents are prone to thrombosis after prolonged exposure [18]

Plancher et al. [19] (2025)	Retrospective study	57,135	4000	No	Yes	High- versus low-altitude patients after total knee arthroplasty (TKA)	VTE incidence was higher in the high-altitude group at 30 days (OR = 1.15) and 90 days (OR = 1.20) [19]

Cancienne et al. [20] (2017)	Retrospective study	458,655	4000	Confirmed, CTPA	Yes	High- vs low-altitude patients receiving meniscectomy/chondroplasty	30-day rates of VTE (OR = 2.0, *p*=0.0003) and PE (OR = 2.5, *p*=0.0099) were significantly higher after procedures at high altitude vs low altitude [20]

**Table 2:** Comparison of venous thromboembolism characteristics between high-altitude and low-altitude regions.

	Deep vein thrombosis at high altitude	Deep vein thrombosis at low altitude
Epidemiology	Studies have shown that among individuals residing continuously at high altitude (> 15,000 feet) for 3-4 months, the incidence of deep vein thrombosis (with or without pulmonary embolism) reaches 2469 per 100,000 person-years [7]	The incidence of deep vein thrombosis ranges from 53 to 162 cases per 100,000 population [31, 32]
Risk factors	High altitude promotes thrombosis via hypoxia, dehydration, stasis, and inherited or acquired prothrombotic factors	Major surgery, severe trauma, prolonged bed rest, limb immobilization, malignancy
Clinical presentation	Limb pain, swelling, and localized redness, warmth, and tenderness may occur, with symptoms potentially overlapping with high-altitude–related joint discomfort	Sudden limb swelling and pain, pitting edema and warmth, calf or thigh tenderness, positive Homans' sign, superficial vein dilation, phlegmasia cerulea dolens, absent peripheral pulse
Lab findings	Elevated hemoglobin and hematocrit due to chronic hypoxia, increased blood viscosity, elevated D-dimer (may reflect both thrombosis and hypoxic baseline), decreased arterial oxygen partial pressure (PaO_2_), normal or mildly elevated platelets and fibrinogen	D-dimer elevated in acute thrombotic events, hemoglobin and hematocrit usually normal, blood viscosity normal, PaO_2_ typically normal, platelets, and fibrinogen may be elevated in inflammatory or malignant conditions
Treatment	Anticoagulation is primary; descent to lower altitude recommended. Thrombolysis used cautiously due to bleeding risk	Standard anticoagulation; thrombolysis/intervention as indicated. Altitude not a treatment concern
Prognosis	Higher risk of recurrence, PE, and complications if hypoxia persists	Generally good with treatment; complications such as post-thrombotic syndrome or PE in severe cases

**Table 3:** Comparison of pulmonary embolism characteristics between high-altitude and low-altitude regions.

	High-altitude pulmonary embolism	Low-altitude pulmonary embolism
Epidemiology	Pulmonary embolism appears more frequent at high altitude, but specific epidemiological data are lacking.	The annual incidence of pulmonary embolism ranges from 39 to 115 cases per 100,000 population [31, 32]
Pathophysiology	Hypoxia-induced polycythemia, hyperviscosity, slow venous return, endothelial dysfunction	Typically caused by thrombus dislodgement from deep veins
Clinical features	Severe dyspnea, marked hypoxemia, sometimes RV failure or overlap with HAPE	Typical PE symptoms: Chest pain, dyspnea, hemoptysis
Lab results	D-dimer↑, Hb↑, Hct↑, significant hypoxemia (↓PaO_2_)	D-dimer↑, mild, or no hypoxemia
Imaging findings	Central or multifocal PE on CTPA, frequent pulmonary hypertension; RV strain on echocardiography	Peripheral PE more common, PAP normal, or slightly elevated
Treatment considerations	Anticoagulation, oxygen therapy, and altitude descent, Thrombolysis when necessary	Anticoagulation, oxygen therapy, and thrombolysis when necessary

**Table 4:** Differential diagnosis and practical diagnostic tools for pulmonary embolism at high altitude.

Differential diagnoses	Typical manifestations	Key features for distinction	Available diagnostic tools at high altitude
High-altitude pulmonary edema (HAPE)	Dyspnea, cough, fatigue, cyanosis, and bilateral pulmonary crackles	Often improves with descent and oxygen; diffuse bilateral infiltrates on chest imaging	Chest X-ray, oxygen challenge test, response to descent/oxygen
Acute coronary syndrome (ACS)	Chest pain, dyspnea, diaphoresis, ECG changes	Elevated cardiac enzymes (troponin), typical ECG ischemic patterns	ECG, troponin test (if available), bedside echocardiography
Community-acquired pneumonia	Fever, cough with sputum, localized lung crackles or rales	Consolidation on chest imaging, elevated WBC count, febrile response	Chest X-ray, WBC count, point-of-care lung ultrasound
Spontaneous pneumothorax	Sudden chest pain and dyspnea, hyperresonance, decreased breath sounds	Absence of lung markings on imaging; tracheal deviation in tension pneumothorax	Chest X-ray, bedside ultrasound
High-altitude cerebral edema (HACE)	Altered mental status, ataxia, headache, nausea, vomiting	Neurological symptoms without focal lung findings; often coexists with HAPE	Neurological exam, clinical context, response to descent, and brain CT (if available)

## Data Availability

This study did not generate any new datasets. All data analyzed are from publicly available sources, as cited in the manuscript.
